# Electrophysiological neuromuscular alterations and severe fatigue predict long-term muscle weakness in survivors of COVID-19 acute respiratory distress syndrome

**DOI:** 10.3389/fneur.2023.1235734

**Published:** 2023-11-22

**Authors:** Marco Benedini, Marta Cogliati, Tea Lulic-Kuryllo, Elena Peli, Stefano Mombelli, Stefano Calza, Bruno Guarneri, Alessandro Cudicio, Andrea Rizzardi, Michele Bertoni, Stefano Gazzina, Stefania Renzi, Nicola Gitti, Frank A. Rasulo, Alberto Goffi, Matteo Pozzi, Claudio Orizio, Francesco Negro, Nicola Latronico, Simone Piva

**Affiliations:** ^1^Department of Clinical and Experimental Sciences, University of Brescia, Brescia, Italy; ^2^Department of Anesthesia, Critical Care and Emergency, ASST Spedali Civili University Hospital, Brescia, Italy; ^3^Department of Medical and Surgical Specialties, Radiological Sciences and Public Health, University of Brescia, Brescia, Italy; ^4^Department of Molecular and Translational Medicine, University of Brescia, Brescia, Italy; ^5^Department of Continuity of Care and Frailty, Neurology Unit, ASST Spedali Civili, Brescia, Italy; ^6^“Alessandra Bono” Interdepartmental University Research Center on LOng-Term Outcome (LOTO) in Critical Illness Survivors, Department of Medical and Surgical Specialties, Radiological Sciences and Public Health, University of Brescia, Brescia, Italy; ^7^Department of Medicine and Interdepartmental Division of Critical Care Medicine, University of Toronto, Toronto, ON, Canada; ^8^St. Michael's Hospital and Li Ka Shing Knowledge Institute, Keenan Research Centre, Unity Health Toronto, Toronto, ON, Canada; ^9^Department of Emergency and Intensive Care, ASST Monza, Monza, Italy; ^10^“Teresa Camplani” Interdepartmental University Research Center on the Neuromuscular Function and the Adapted Motor Activity, Department of Clinical and Experimental Sciences, University of Brescia, Brescia, Italy

**Keywords:** acute respiratory distress syndrome, COVID-19, muscle weakness, fatigue, electrical neuromuscular function

## Abstract

**Introduction:**

Long-term weakness is common in survivors of COVID-19-associated acute respiratory distress syndrome (CARDS). We longitudinally assessed the predictors of muscle weakness in patients evaluated 6 and 12 months after intensive care unit discharge with in-person visits.

**Methods:**

Muscle strength was measured by isometric maximal voluntary contraction (MVC) of the tibialis anterior muscle. Candidate predictors of muscle weakness were follow-up time, sex, age, mechanical ventilation duration, use of steroids in the intensive care unit, the compound muscle action potential of the tibialis anterior muscle (CMAP-TA-S100), a 6-min walk test, severe fatigue, depression and anxiety, post-traumatic stress disorder, cognitive assessment, and body mass index. We also compared the clinical tools currently available for the evaluation of muscle strength (handgrip strength and Medical Research Council sum score) and electrical neuromuscular function (simplified peroneal nerve test [PENT]) with more objective and robust measures of force (MVC) and electrophysiological evaluation of the neuromuscular function of the tibialis anterior muscle (CMAP-TA-S100) for their essential role in ankle control.

**Results:**

MVC improved at 12 months compared with 6 months. CMAP-TA-S100 (*P* = 0.016) and the presence of severe fatigue (*P* = 0.036) were independent predictors of MVC. MVC was strongly associated with handgrip strength, whereas CMAP-TA-S100 was strongly associated with PENT.

**Discussion:**

Electrical neuromuscular abnormalities and severe fatigue are independently associated with reduced MVC and can be used to predict the risk of long-term muscle weakness in CARDS survivors.

## 1 Introduction

Intensive care unit (ICU) patients surviving critical illness may suffer prolonged physical, cognitive, and mental health impairments, collectively known as “post-intensive care syndrome” (1–4). Physical function is affected in 20%−80% of ICU survivors and significantly affects the quality of life, independence in activities of daily living, and the return to work (4–6). Physical function impairments manifest with reduced limb strength and range of motion, modified proprioception and balance, pain (7, 8), fatigue (9), activity limitations, and restrictions on participation in social contexts (10) and are common in COVID-19 ICU survivors (11–22). ICU-acquired muscle weakness is described in 43% (interquartile range 25%−75%) of critically ill patients (23) and is a major predictor of long-term weakness (24). Post-hospital predictors of long-term weakness have not been explored.

In a previous study of COVID-19-associated acute respiratory distress syndrome (CARDS) survivors (11), we found that handgrip strength (HGS) assessed with dynamometry was 70% of the predicted normal value at 3 months and was significantly improved over time. Simplified electroneurography of the peroneal nerve (PENT) (25) showed a critical illness, polyneuromyopathy, in 23 of 59 patients (39%). However, global muscle strength assessed using the Medical Research Council (MRC) sum score (MRCss) found significant weakness (MRCss <48) in only three patients at 3 months and in one patient at 6 and 12 months. The MRCss, despite its ability to quickly identify muscle weakness, is influenced by subjective judgment, leading to variability and potential bias, particularly in follow-up assessments conducted by different operators. MRCss has other limitations, including a ceiling effect that prevents the detection of milder forms of muscle weakness, and it does not account for factors such as muscle length and shortening velocity, which can significantly affect muscle force generation capacity. To overcome these limitations, it is essential to use objective measures of force in muscle groups relevant to daily life activities, such as standing and walking.

Maximum voluntary contraction (MVC) serves as an excellent alternative in this regard; MVC is based on an objective value that remains uninfluenced by the operator's subjective perception, ensuring measurement reproducibility over time and enabling accurate monitoring of changes in muscle strength. Furthermore, MVC can detect even subtle variations in strength, increasing its sensitivity.

Similarly, PENT focuses on the evaluation of nerve function in specific muscles of the foot that may not be associated with the ankle movements that are more relevant for individual and functional independence. Robust electrophysiological measures (e.g., compound muscle action potential, CMAP) of targeted muscles that are important for daily life activities may be more appropriate for evaluating the rate of neural impairment of specific muscle groups.

The aims of this study were (1) to identify the post-hospital predictors of long-term muscle strength, as measured by isometric MVC of the tibialis anterior (TA) muscle, and (2) to compare the clinical tools currently available for evaluating neuromuscular function (HGS, MRCss, and PENT) using objective and robust measures of force (MVC) and electrophysiological evaluation of nerve function (CMAP) on the TA muscle for its essential role in ankle control.

## 2 Methods

We conducted an observational longitudinal study of adult (≥18 years old) ARDS survivors with confirmed SARS-CoV-2 infection admitted to the ICUs of the ASST Spedali Civili University Hospital of Brescia, Italy, from 25 February 2020 to 17 November 2021. In this study, we used data from our follow-up clinic that was founded in 2014 and partnered in 2020 by a research center on LOng-Term Outcomes (called LOTO) in critical illness survivors (26). The LOTO database contains data from 2014 and continuously records data on patients visited at the follow-up clinic.

ARDS was diagnosed according to the Berlin criteria, and all patients received invasive mechanical ventilation. The Ethics Committee of Brescia approved this study (study title: The PIC syndrome: follow-up of the intensive care patient; approval number: NP3369; approval date: 11 December 2018), and written informed consent was obtained from all participants (or substitute decision-makers) before data collection. The study was carried out according to the Declaration of Helsinki of 1975 and the EU GCP-ICH Guidelines. Patient demographic and clinical characteristics at ICU admission were obtained from hospital records. We adhered to the STROBE reporting guidelines (27). The present study was registered at ClinicalTrial.gov (NCT: NCT04608994).

### 2.1 Follow-up protocol

Patients were invited to attend a post-ICU clinic, where a standardized assessment of physical, cognitive, and mental health status was performed for each patient at 6 and 12 months after ICU discharge. A detailed presentation of the protocol has been published elsewhere [see appendix of (11)].

*Neuromuscular function* was assessed with MVC, HGS, MRCss, CMAP of the TA (CMAP-TA-S100), and PENT. We also assessed fatigue and mental and cognitive variables because we hypothesized that they could influence muscle strength.

**Figure 1:**
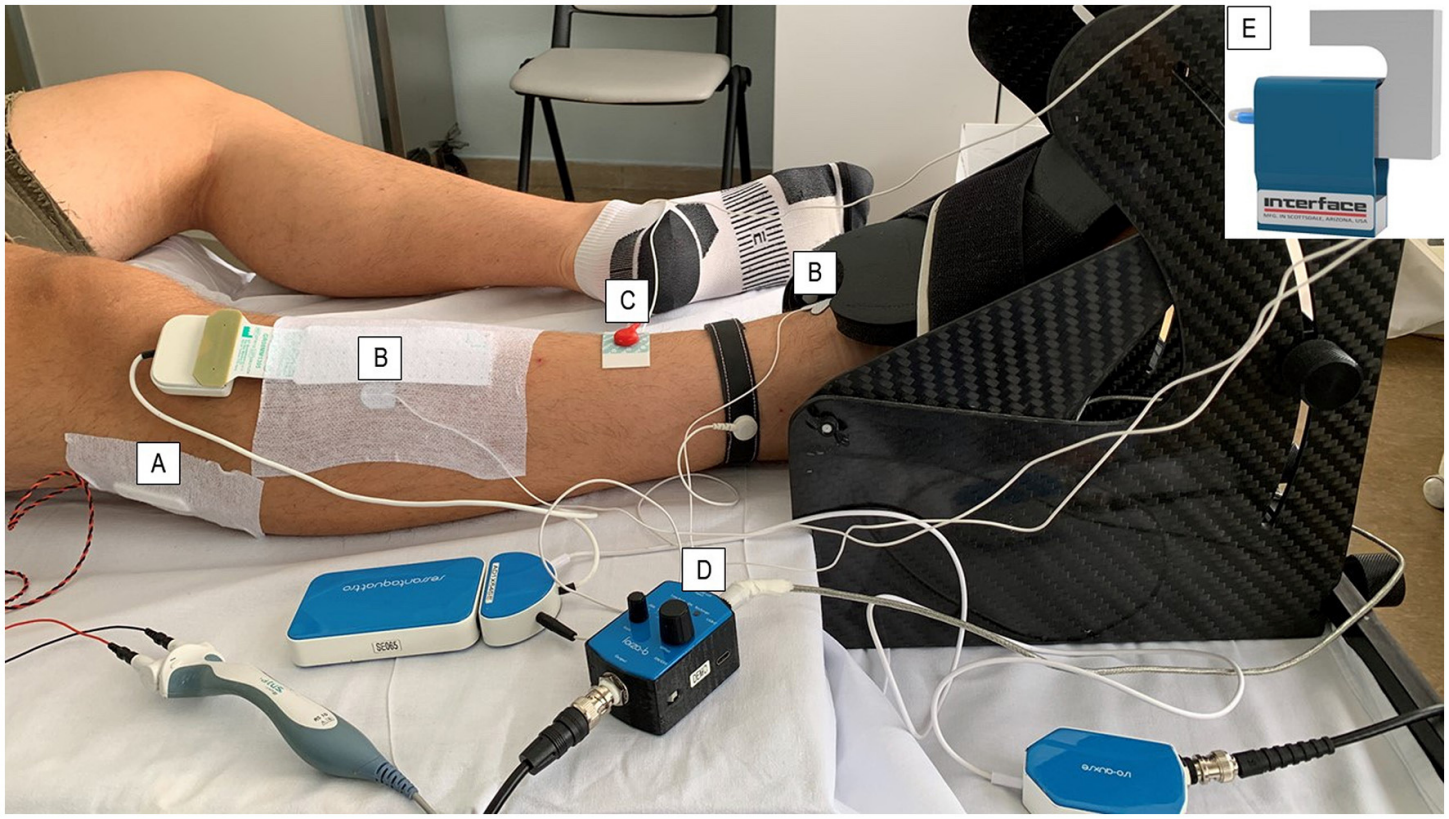
Follow-up protocol set-up. The patients were asked to position their dominant leg in a carbon ankle ergometer, and the foot was supported with velcro straps. The common peroneal nerve was stimulated under the peroneal head with a **(A)** bar stimulator using the CMAP scan technique. **(B)** Surface electrodes were placed on the belly and distally on the tendon of the TA muscle to identify the CMAP-TA-S100 value. The **(C)** ground electrode was placed on the ankle of the same leg to prevent interference with biopotential signals. **(D)** The force amplifier amplified the force signal detected by **(E)** the load cell during both stimulated contraction and maximal voluntary contraction.

Muscle strength was primarily assessed with the measurement of the MVC (lower limb dynamometry; Figure 1). Briefly, patients were asked to perform maximal isometric ankle dorsiflexion with their dominant leg. The foot was strapped to the plate of a custom-made carbon dynamometer equipped with a load cell (model SM-100N) to measure the applied tension during ankle dorsiflexion. The knee was fully extended (180°), with the ankle placed in a neutral position (110°) (28). Patients performed three MVCs in dorsiflexion of the foot with a 1.5-min rest between each trial. Each trial allowed 3 s to reach the maximal contraction, which was maintained for another 3 s. During the trial, researchers verbally encouraged participants, and the maximal force recorded was used as a measure of MVC.

HGS was measured using a dynamometer (upper limb dynamometry). Three repetitions were performed, and the maximal value was used as a measure of HGS. We also reported the HGS as a percentage of the predicted normal value standardized per age and sex (29).

In addition, we assessed the MRCss, which provides a global measure of muscle strength. An MRCss of <48 indicated significant weakness (30). We also considered an MRCss of ≤ 55 to indicate mild weakness (31).

Electrical neuromuscular function was assessed by measuring the CMAP of the deep peroneal nerve at the level of the TA muscle (CMAP-TA) and of the extensor digitorum brevis muscle (PENT). The PENT, obtained by the minimum stimulation amplitude that evokes the maximal activation of the extensor digitorum brevis muscle, was considered abnormal if the amplitude was <5.26 mV in both legs (25, 32). The CMAP-TA, which was obtained at the level of the TA muscle to better correlate the strength performance measured with the isometric ankle dorsiflexion, was recorded using a novel CMAP scan application on a Viking Select EMG system (CareFusion, San Diego, CA; Figure 1). Briefly, CMAP-TA was obtained from the TA of the dominant leg using surface electrodes. The negative and positive electrodes were placed on the TA belly and distally on the tendon, respectively, and the ground electrode was placed at the ankle of the same leg. The leg positioning was the same as for the previously described MVC. The common peroneal nerve was stimulated under the peroneal head with a bar stimulator (Spes Medica Srl, Genoa, Italy) with an interelectrode distance of 2.5 cm while patients remained fully relaxed. The stimulation was started at 0 mA and then was increased by 0.1 mV in intensity. Two thresholds were recorded: (1) the minimum stimulation intensity needed to obtain a visible action potential (S0) and (2) the minimum stimulation intensity needed to elicit a maximal response from the TA (S100). The detection of these two threshold values allowed the identification of the interval within which 500 stimuli (frequency 2 Hz and duration 0.2 ms) were applied in decrements from S100 to S0. The action potential amplitude in millivolts obtained at S100 (CMAP-TA-S100) was used as the main measure of neuromuscular function. PENT is a simplified neurophysiological technique with high sensitivity (100%) and good specificity (85%) that has been validated as a screening test for critical illness polyneuropathy and myopathy (25, 32).

Activity limitation was evaluated by the 6-min walk test (6MWT) as a performance-based measure and the fatigue severity score (FSS). For 6MWT, predicted values were calculated according to Enright et al. (33). Self-reported fatigue was assessed using the Fatigue Severity Score (FSS), a 9-item scale with questions about how fatigue has affected the person's activities and lifestyle during the past 2 weeks. An FSS score of ≥36 indicated severe fatigue (34).

*Mental health assessment* was performed by administering (1) the Hospital Anxiety and Depression Scale (HADS) questionnaire, on which a score ≥8 for both subdomains of depression and anxiety was considered abnormal (35), and (2) *Cognition* was assessed using the Montreal Cognitive Assessment (MoCA), a short cognitive screening tool that has been validated as a general cognitive screening test (22, 36).

### 2.2 Statistical analyses

Quantitative variables were described with means ± standard deviations (SD) or median ± interquartile range (IQR), while categorical variables were summarized with counts and percentages. We assessed the normality of the variables using the Shapiro-Wilk test. The relationship between the measured physical performance variables and the follow-up times (6 and 12 months) was modeled using linear mixed models (LMMs) or generalized LMMs, as appropriate. All models were fit while assuming participants as the random intercept and follow-up visit time as a fixed effect.

MVC was modeled using LMM with random intercepts (participants). The final models were defined using a backward variable selection based on the Akaike Information Criterion (AIC), starting from a full model that included the following variables as candidate predictors: follow-up time, sex, age, body mass index (BMI), mechanical ventilation duration, use of steroids in the ICU, SAPS II, 6MWT, CMAP-TA-S100, severe fatigue using the FSS, HADS scores for depression and anxiety, the presence of cognitive impairment using the MoCA scale, and all pairwise interaction terms between follow-up time and all variables included in the model. The formula for the model was as follows:


$$\begin{array}{l} \text{MVC }\sim \;1\;+\text{ follow up time }+\text{ variables }+\;\left(1\;|\text{ record\_id}\right) \end{array}$$

To analyze the correlations between MVC, HGS, and MRCss and between CMAP-TA-S100 and PENT, we used LMM with a random intercept (participant). All tests were two-sided, and a *P*-value of < 0.05 was considered statistically significant. No data imputation was performed, and all analyses were conducted using R (version 4.1.1).

## 3 Results

A total of 52 patients, 38 (73.1%) men, were enrolled in the study and visited at 6 and 12 months. The demographic and clinical characteristics of patients during their ICU stay are presented in Table 1.

**Table 1:** Demographic characteristics and ICU variables of patients enrolled in the study.

	**6 months (*N* = 52)**
Sex, men, *N* (%)	38 (73.1%)
Age (years), mean (SD)	61.3 (8.75)
BMI at ICU admission (kg/m^2^), mean (SD)	28.3 (3.58)
SAPS II, mean (SD)	30.3 (9.37)
Use of NIV pre-ICU, *N* (%)	36 (69.2%)
Duration of mechanical ventilation (days), mean (SD)	11.7 (15.1)
Pronation, *N* (%)	25 (48.1%)
Tracheostomy, *N* (%)	17 (32.7%)
Use of steroids, *N* (%)	37 (71.2%)
Catecholamines, *N* (%)	21 (40.4%)
**Comorbidities**	
No comorbidity	5 (9.6%)
1 comorbidity	18 (34.6%)
2 comorbidities	11 (21.2%)
3 comorbidities	1 (1.9%)
≥4 comorbidities	5 (9.6%)
ICU LOS (days), mean (SD)	16.2 (17.0)
H LOS (days), mean (SD)	37.8 (29.0)

SD, standard deviation; BMI, body mass index; SAPS II, simplified acute physiology score; NIV, non-invasive ventilation; iNO; ICU, intensive care unit; H, hospital; LOS, length of stay.

Missing values [number of patients (percentage)]: SAPS II: 12 (23.1%); use of pre-ICU admission NIV: 3 (5.8%); mechanical ventilation duration: 3 (5.8%); pronation: 3 (5.8%); tracheostomy: 6 (11.5%); steroids: 13 (25.0%); catecholamine: 4 (7.7%); comorbidities: 12 (23.1%).

**Figure 2:**
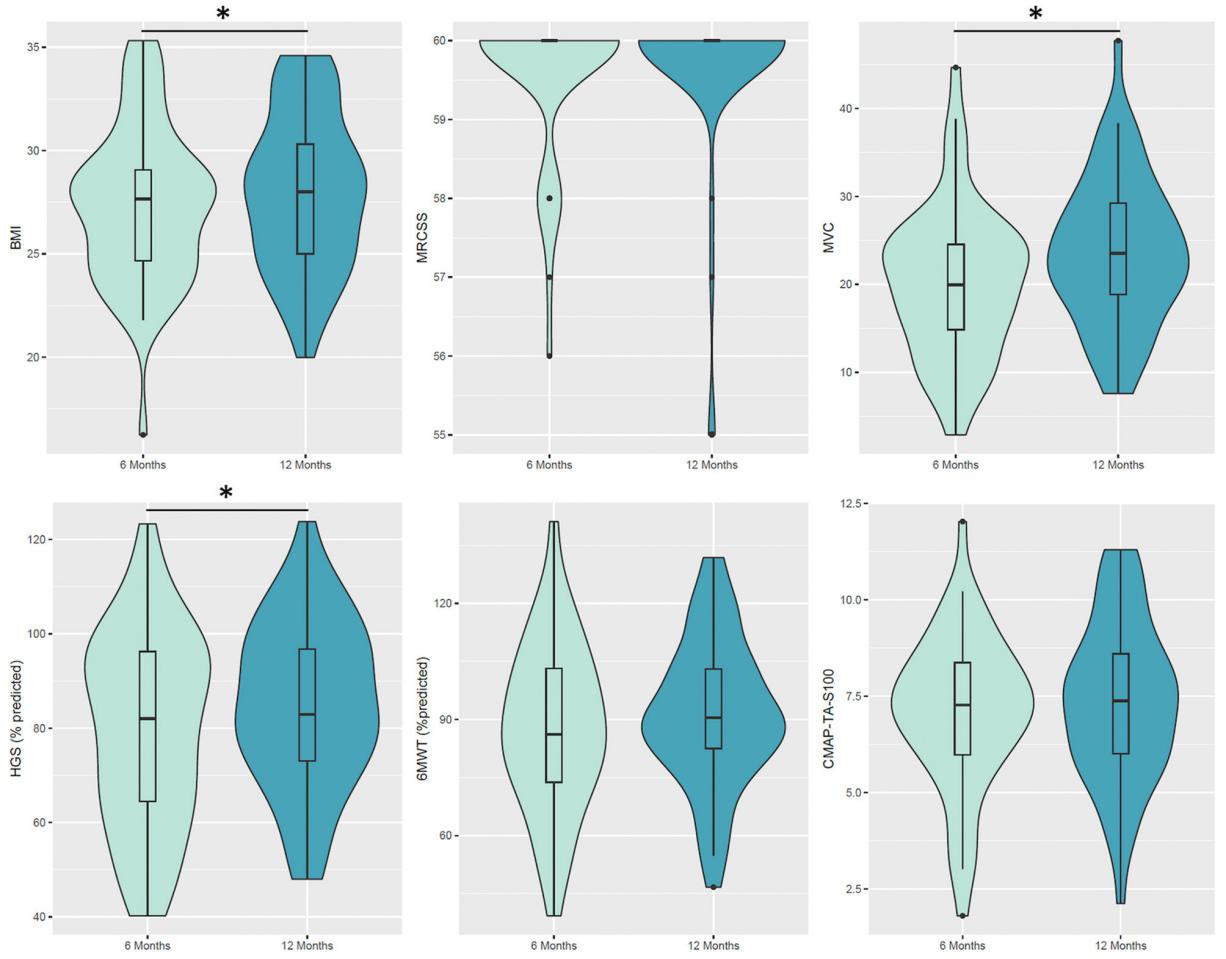
Violin plot for the considered variables at 6 and 12 months. Boxes indicate the first and third quartiles. Black thick lines in the bar graphs denote the median value. The whiskers extending from the box show the range of the data, excluding outliers. Patients exhibited significant changes from 6 to 12 months in BMI (*p*-adjusted < 0.001), MVC (*p*-adjusted = 0.003) and HGS (% predicted, *p*-adjusted < 0.001). BMI, body mass index; MRCss, Medical Research Council Sum Score; MVC, maximal voluntary contraction; HGS, handgrip dynamometry; 6MWT, 6-min walk test; CMAP, the compound muscle action potential; TA, tibialis anterior.

Muscle strength improved over time. MVC improved at 12 months [estimate difference (ED) = 3.43 kg when compared with 6 months, *P*-adjusted = 0.003). HGS improved at 12 months as both absolute values in kilograms and percentage predicted value (ED =5.39%, *P*-adjusted < 0.001). An MRCss was ≥48 in all patients. CMAP-TA-S100 (ED 0.4 mV, *P*-adjusted = 0.106) did not improve at 12 months, whereas PENT (ED 1.19 mV, *P*-adjusted = 0.003) improved at 12 months (Table 2, Figure 2). Severe fatigue was reported by 30.8% at 6 months and 21.2% at 1 year, without significant improvement over time. Cognitive impairment was present in a significant proportion of patients (21.2% at 6 months and 15.4% at 12 months).

**Table 2:** Summary of physical, mental health, and cognitive function variables and neuromuscular electrophysiological measurements at 6 and 12 months.

	**6 months (*N* = 52)**	**12 months (*N* = 52)**	**Estimated difference (95% CI)**	***P*-value**	***P*-adjusted^d^**
BMI (kg/m^2^), mean (SD)	27.4 (3.79)	28.0 (3.74)	0.61 (0.27 to 0.96)	0.001	<0.001
MRCss^a^, median (IQR)	60 (60–60)	60 (60–60)	0.11 (−0.24 to 0.56)	0.533	0.630
MVC (kg), mean (SD)	20.2 (8.55)	23.6 (8.41)	3.43 (2.05 to 4.82)	<0.001	0.003
Dominant hand grip strength (kg), mean (SD)	30.5 (10.4)	32.4 (9.72)	1.95 (0.928 to 2.96)	<0.001	<0.001
Dominant hand grip strength (% predicted)^b^, mean (SD)	79.7 (20.5)	85.0 (17.6)	5.39 (2.71 to 8.07)	<0.001	<0.001
6MWT, mean (SD)	450 (108)	474 (96.1)	24.0 (−4.89 to 52.8)	0.096	0.156
6MWT, (%predicted)^c^, mean (SD)	87.9 (21.8)	91.2 (19.0)	4.41 (−1.46 to 10.3)	0.132	0.190
CMAP-TA-S100 (mV), mean (SD)	7.09 (1.99)	7.36 (2.07)	0.4 (0.02 to 0.8)	0.049	0.106
PENT (mV), mean (SD)	6.21 (4.80)	6.82 (5.21)	1.19 (0.52 to 1.86)	0.001	0.003
Fatigue (Fatigue Severity Score ≥36), *N* (%)	16 (30.8%)	11 (21.2%)	−3.0 (−0.31 to −6)	0.076	0.141
Presence of depression (HADS >7), *N* (%)	10 (19.2%)	6 (11.5%)	−15.2 (−24.1 to −1.18)	0.781	0.831
Presence of anxiety (HADS >7), *N* (%)	7 (13.5%)	10 (19.2%)	0.138 (−1.13 to 1.4)	0.831	0.831
Cognitive impairment (MoCA <26), *N* (%)	11 (21.2%)	8 (15.4%)	−1.45 ( −3.59 to 0.69)	0.185	0.241

BMI, body mass index; SD, standard deviation; MVC, maximal voluntary contraction; MRCss, Medical Research Council Sum Score; CMAP, the compound muscle action potential; TA, tibialis anterior; PENT, PEroneal Nerve Test; HADS, Hospital Anxiety and Depression Scale; MoCA, Montreal Cognitive Assessment.

Missing value (6–12 months): MRCss: 9 (17.3%) to 2 (3.8%); HADS: 12(23.1%) to 0 (0%); PTSD: 0 (0%) to 2 (3.8%); MoCA: 3 (5.8%) to 0 (0%).

^a^None of the patients had an MRCss <55.

^b^Calculated using established reference values provided by Gilbertson et al. (29).

^c^Predicted value for the 6MWT was calculated according to Carenzo et al. (18) and Enright et al. (33).

^d^P-adjusted using Benjamini and Hochberg (BH) correction.

**Figure 3:**
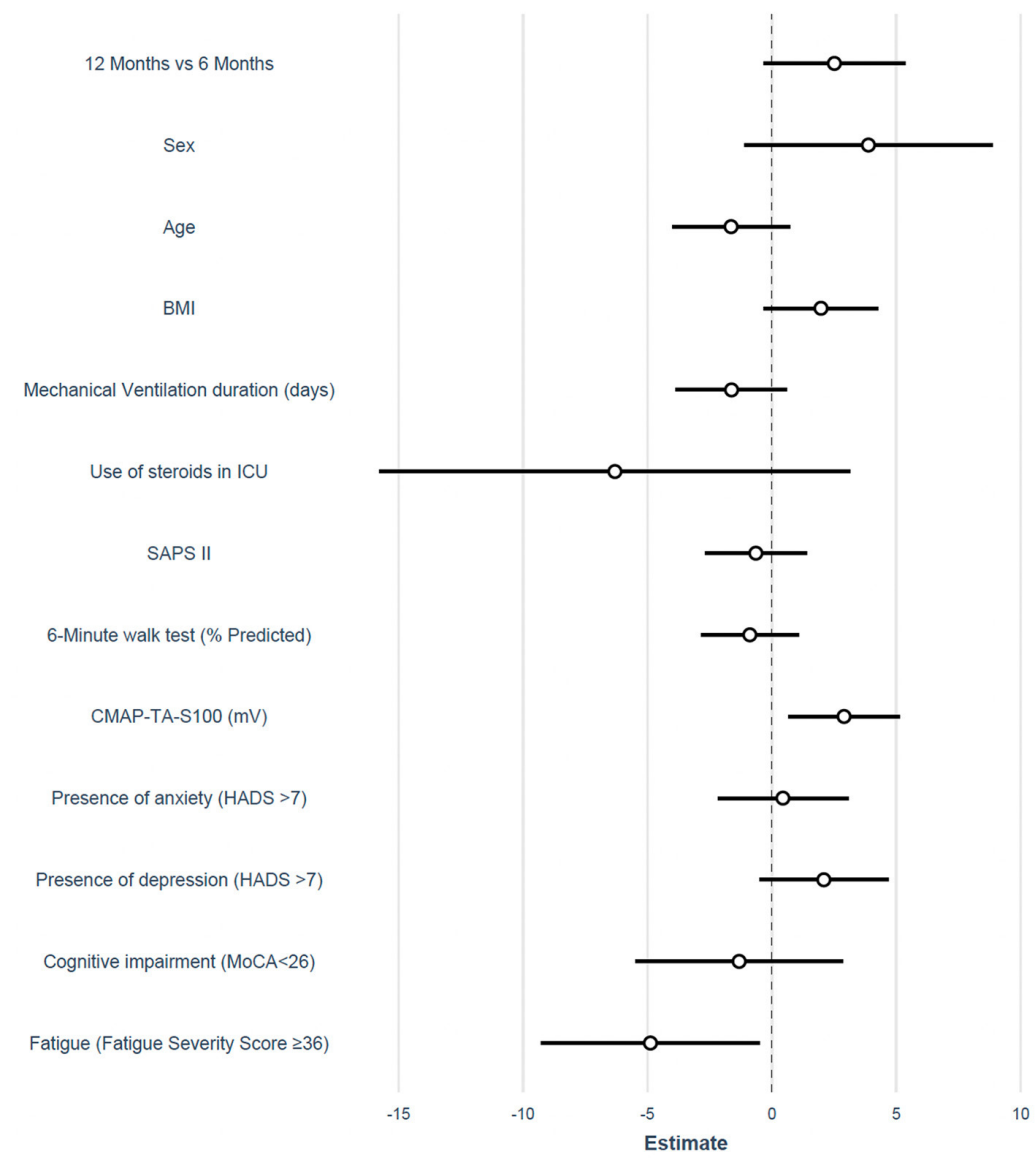
Forest plot of the estimated difference for the adjusted mixed model on MVC. BMI, body mass index; ICU, intensive care unit; SAPS II, simplified acute physiology score; CMAP, the compound muscle action potential; TA, tibialis anterior; HADS, hospital anxiety and depression scale; MoCA, Montreal cognitive assessment.

Multivariable analysis showed that MVC was independently associated with CMAP-TA-S100 (ED 1.4 kg for each millivolt increase in CMAP-TA-S100, *P* = 0.016) and fatigue (ED −4.88 kg in patients with fatigue, *P* = 0.036). There was no interaction between follow-up time and the selected variables (Table 3, Figure 3).

**Table 3:** Effect estimates and corresponding 95% confidence intervals (CIs) for *muscle strength* prediction using maximal voluntary contraction (MVC in kg) computed using a linear mixed model (LMM) with random intercept.

**Coefficient**	**Model MVC (kg)**		
	**Estimates**	**95% CI**	***P*** **-value**
12 months vs. 6 months	2.51	−0.43 to 5.45	0.092
Sex (men)	3.88	−1.29 to 9.05	0.137
Age (years)	−0.18	−0.45 to 0.09	0.184
Body mass index (kg/m^2^)	0.54	−0.11 to 1.19	0.103
Mechanical ventilation duration (days)	−0.09	−0.21 to 0.04	0.164
Use of steroids in ICU	−6.31	−16.09 to 3.48	0.200
SAPS II	−0.06	−0.27 to 0.14	0.538
6MWT (%predicted)	−0.05	−0.15 to 0.06	0.382
CMAP-TA-S100 (mV)	1.40	0.28 to 2.52	**0.016**
Presence of anxiety (HADS >7)	0.12	−0.63 to 0.87	0.743
Presence of depression (HADS >7)	0.52	−0.15 to 1.19	0.122
Cognitive impairment (MoCA <26)	−1.32	−5.63 to 2.99	0.540
Fatigue (Fatigue Severity Score ≥36)	−4.88	−9.43 to −0.34	**0.036**
Marginal *R*^2^/conditional *R*^2^	0.579/0.778		

The variables included in the final model were selected using a backward procedure using the AIC criterion.

CIs, confidence intervals; SAPS II, Simplified Acute Physiology Score; CMAP, Compound Muscle Action Potential amplitude; TA, tibialis anterior; HADS, Hospital Anxiety and Depression Scale; MoCA, Montreal Cognitive Assessment. The bold values represent statistically significant results (*P*-value < 0.05).

MVC was strongly associated with HGS (ED 0.41 kg (95% confidence intervals: 0.26–0.56) increase in HGS for each 1-kg increase in MVC, *P* < 0.001) but not with an MRCss (ED = 0.25, *P* = 0.712). CMAP-TA-S100 was strongly associated with PENT (ED 0.21 mV increase in PENT for each millivolt increase in CMAP-TA-S100, *P* < 0.001). Patients with abnormal PENT (<5.26 mV) had a mean (SD) CMAP-TA-S100 of 6.87 (1.94) mV, whereas patients with normal PENT had a mean (SD) CMAP-TA-S100 of 7.95 (1.83) mV.

## 4 Discussion

In this longitudinal, 1-year study of CARDS survivors, we found that muscle weakness in COVID-19 patients improved at 12 months and was associated with electrical neuromuscular dysfunction (measured using CMAP-TA-S100) and severe fatigue. Moreover, we found a strong correlation between HGS and lower limb dynamometry (MVC) and between CMAP-TA-S100 and PENT.

MVC was independently associated with CMAP amplitudes measured from the TA muscle: muscle strength increased by 1.4 kg for each millivolt increase in CMPA-TA-S100 amplitude. These findings indicate that CARDS patients have abnormal CMAP-TA-S100 and thus impairments in MVC generation, with improvements observed at 1-year post-hospital discharge. Reduced MVC has previously been reported in quadriceps and biceps brachii in COVID-19 survivors (11).

MVC was independently associated with severe fatigue (patients with fatigue had a mean MVC of 5 kg lower than patients without fatigue). Fatigue was present in 30% of our patients at 6 months without a significant improvement over time, in line with our previous result (37, 38), and two recent meta-analyses on post-COVID fatigue (39). To the best of our knowledge, this is the first study to demonstrate that severe fatigue predicts muscle weakness in critically ill survivors. Post-COVID fatigue, defined as an overwhelming and sustained subjective sense of physical, emotional, and/or cognitive exhaustion that is not related to recent physical activity (40), has been associated with a distinct pattern of pathological brain changes involving the thalamus and the basal ganglia (41), which support important cognitive functions such as memory, motivation, and reward-guided behavior, among a wide range of functions in addition to motor control. Our findings that the persistent subjective experience of fatigue is related to peripheral measures of physical performance may have implications for future treatments, such as self-guided or health professional-guided physical and cognitive interventions (25, 32).

The strong association between CMAP-TA-S100 and PENT amplitudes is important for two reasons. First, it confirms that electrical neuromuscular alterations are diffuse so that the recording site when a peroneal nerve is stimulated (i.e., TA versus extensor digitorum brevis) does not lead to differences in diagnostic findings. Second, CMAP-TA-S100 requires specialized personnel and instruments, and values in a normal population are not available. In contrast, the PENT is a rapid screening test that has been validated in multi-center studies (42) and can be quickly administered. Despite a strong association between a risk factor (i.e., altered electrical neuromuscular activity assessed with CMAP-TA-S100 or PENT) and the disease outcome (muscle weakness), not every predictor is a cause (43), and validation studies are needed before the altered electrical neuromuscular function can be considered causally related to muscle weakness in CARDS survivors.

HGS was strongly associated with MVC measured from the TA muscle (for each 0.5-kg increase in HGS, there was a 1-kg increase in MVC, *P* < 0.001), suggesting that HGS is a representative of global muscle strength in CARDS survivors and might serve as a quick screening tool for repeated muscle strength assessment during follow-up (43). However, further studies are needed to validate the diagnostic accuracy of HGS compared with MVC in a new cohort of patients.

The MRCss was mostly normal, regardless of the timing of assessment and despite abnormalities in MVC, HGS, and electrophysiological parameters. This result confirms that the MRCss misses an important group of CARDS patients with milder weakness at long-term follow-up.

The study limitations should be considered in the interpretation of our results. This study was conducted at a single center, and the findings need to be externally validated in an independent cohort. Patients were followed up for 1 year, but assessment at all time points for all patients was not possible because of restricted hospital access and patient's unwillingness to continue participation in the study. Moreover, the study was conducted in patients with CARDS, and the generalization to patients with classic ARDS is not possible, although plausible. Finally, the associations we found do not imply causality.

## 5 Conclusion

Electrical neuromuscular abnormalities (CMAP-TA-S100) and the presence of severe fatigue were independently associated with reduced MVC and can be used to predict the risk of long-term muscle weakness in CARDS survivors.

## Data availability statement

The raw data supporting the conclusions of this article will be made available by the authors, without undue reservation.

## Ethics statement

The studies involving humans were approved by Comitato Etico di Brescia e Provincia. The studies were conducted in accordance with the local legislation and institutional requirements. The participants provided their written informed consent to participate in this study.

## Author contributions

MBen, MC, and TL-K: conceptualization, methodology, investigation, formal analysis, writing—original draft, writing—review and editing, and visualization. EP, BG, MBer, SG, SR, NG, and FR: investigation, visualization, and writing—review and editing. SM: writing—review and editing and visualization. SC and AG: formal analysis, writing—review and editing, and visualization. AC and AR: investigation, writing—review and editing, and visualization. MP: investigation, visualization, and writing review and editing. CO: conceptualization, writing—original draft, writing—review and editing, and visualization. FN and NL: conceptualization, methodology, writing—original draft, writing—review and editing, and visualization. SP: conceptualization, methodology, formal analysis, writing—original draft, writing—review and editing, and visualization. All authors contributed to manuscript revision, read, and approved the submitted version.
